# Combining Chk1/2 Inhibition with Cetuximab and Radiation Enhances *In Vitro* and *In Vivo* Cytotoxicity in Head and Neck Squamous Cell Carcinoma

**DOI:** 10.1158/1535-7163.MCT-16-0352

**Published:** 2017-01-30

**Authors:** Ling Zeng, Reena R. Beggs, Tiffiny S. Cooper, Alice N.Weaver, Eddy S.Yang

**Affiliations:** 1 Department of Radiation Oncology, University of Alabama at Birmingham School of Medicine, Birmingham, Alabama; 2 Department of Pharmacology and Toxiology, University of Alabama at Birmingham School of Medicine, Birmingham, Alabama; 3 Department of Cell, Developmental, and Integrative Biology, University of Alabama at Birmingham School of Medicine, Birmingham, Alabama; 4 Comprehensive Cancer Center, University of Alabama at Birmingham School of Medicine, Birmingham, Alabama

## Abstract

EGFR inhibition and radiotherapy are potent inducers of DNA damage. Checkpoint kinases 1 and 2 (Chk1/2) are critical regulators of the DNA-damage response, controlling cell-cycle checkpoints that may permit recovery from therapy-associated genomic stress. We hypothesized that Chk1/2 inhibition (CHKi) with prexasertib may enhance cytotoxicity from EGFR inhibition plus radiotherapy in head and neck squamous cell carcinoma (HNSCC). In this study, we found that the addition of CHKi to the EGFR inhibitor cetuximab with and without radiotherapy significantly decreased cell proliferation and survival fraction in human papillomavirus virus (HPV)-positive and HPV-negative HNSCC cell lines. Reduced proliferation was accompanied by decreased checkpoint activation, induced S-phase accumulation, persistent DNA damage, and increased caspase cleavage and apoptosis. Importantly, a significant tumor growth delay was observed *in vivo* in both HPV-positive and HPV-negative cell line xenografts receiving triple combination therapy with CHKi, cetuximab, and radiotherapy without a concomitant increase in toxicity as assessed by mouse body weight. Taken together, the combination of CHKi with cetuximab plus irradiation displayed significant antitumor effects in HNSCCs both *in vitro* and *in vivo,* suggesting that this combination therapy may increase clinical benefit. A clinical trial to test this treatment for patients with head and neck cancer is currently ongoing (NCT02555644).

## Introduction

Head and neck squamous cell carcinomas (HNSCC) are aggressive tumors with high recurrence rates and poor 5-year survival. Although HNSCCs account for only 3% of all cancers in the United States, the incidence of oropharyngeal squamous cell carcinoma (OPSCC) specifically has been increasing over the past 20 years (1). This increase is being driven by the rising prevalence of human papillomavirus virus (HPV)–associated tumors, which are characterized by improved outcomes and increased sensitivity to DNA-damaging therapies such as irradiation and chemotherapy (2, 3). Although HPV is the strongest individual prognostic marker for HNSCC, patient survival is also closely associated with expression of EGFR. EGFR is a cell surface receptor tyrosine kinase that regulates cell proliferation, differentiation, and DNA-damage response and repair (4–6). EGFR is overexpressed or otherwise activated in 90% to 95% of HNSCCs, and contributes to decreased radiosensitivity and poor survival (5). Importantly, EGFR inhibition with the monoclonal antibody cetuximab (C225) in combination with radiotherapy has been shown to increase locoregional control and survival in HNSCC patients (4). Although cetuximab plus radiotherapy is now a standard of care in the treatment of HNSCC, the large majority of patients have intrinsic or acquired resistance to this therapy indicating additional strategies are needed for patients with HNSCC.

One effect of treatment with cetuximab and irradiation is the induction of replication stress and DNA damage with simultaneous suppression of DNA repair (7). These events activate cell-cycle checkpoints, including the serine/threonine kinases Checkpoint 1 and 2 (Chk1/2), resulting in cell-cycle arrest. During this period, cells stabilize replication origins and repair DNA damage before reentering the cell cycle. Although cell-cycle checkpoints are a necessary component of the DNA-damage response in normal cells, they may also be a mechanism by which tumors avoid treatment-induced apoptosis and acquire resistance to EGFR-targeted agents (8). This is especially true of HNSCC, where Chk1 and Chk2 are among the most significantly elevated phosphoproteins in tumors as compared to healthy tissue (9). Moreover, in pancreatic or breast cancer models, the combination of EGFR inhibition, DNA-damage response inhibitors, and irradiation therapy have exhibited synergy (10–12).

A new class of targeted anticancer agents has been developed that inhibits Chk1/2 (CHKi), blocking cell-cycle checkpoint activation, and permitting cell-cycle progression despite unrepaired DNA damage (13). Specifically, the CHKi prexasertib mesylate monohydrate (Eli Lilly) has the added benefit of generating additional double-stranded DNA breaks while simultaneously blocking RAD51-mediated DNA-damage repair (14). This catastrophic combination of effects eventually leads to cell death, and single-agent treatment with prexasertib has been shown to induce persistent DNA damage and significant growth inhibition in cancer cell lines and tumor xenografts (14).

On the basis of these observations, we hypothesized that prexasertib may increase the efficacy of cetuximab plus radiotherapy in HNSCCs. We conducted an *in vitro* and *in vivo* analysis of combination therapy with cetuximab, prexasertib, and irradiation (IR) in HNSCC cell lines. The combination of prexasertib and cetuximab with or without IR inhibited cell proliferation greater than single-agent treatment alone in both HPV-positive and HPV-negative HNSCC cell lines *in vitro*. This effect was associated with induction of apoptosis, persistent DNA damage, and alteration of cell-cycle distribution. On the basis of these in vitro results, we designed *in vivo* studies using xenograft models to test the potential antitumor effects of the combination therapy of prexasertib, cetuximab, and IR. Importantly, triple combination treatment significantly delayed tumor growth in vivo in HNSCC cell line xenografts. These results suggest that prexasertib has activity against head and neck cancer cells, and combining prexasertib, cetuximab, and IR in HNSCC may provide additional clinical benefit and offer a potential therapeutic strategy for this disease.

## Materials and Methods

### Cell culture and reagents

The HPV-negative UM-SCC1, UM-SCC2, and UM-SCC6 cell lines were obtained courtesy of Dr. Thomas E. Carey (2010; University of Michigan, Ann Arbor, MI). HPV-positive UM-SCC47 and UPCI:SCC090 cells were a gift from Dr. Susan Golin (University of Pittsburgh, Pittsburgh, PA) and Dr. John H. Lee (2011; Sanford Cancer Research Center, Sioux Falls, SD). UM-SCC1-luciferase was obtained from Dr. Eben Rosenthal (2011; Stanford University, Stanford, CA). These cell lines have been previously described (12–14). The HPV-negative FaDu (HTB-43) cell line was purchased from the ATCC (2001). UM-SCC1, UM-SCC2, UM-SCC6,UM-SCC47 and UPCI:SCC090 cell lines were maintained in DMEM growth medium (Sigma) supplemented with 10% FBS (SAFC Biosciences) and 1% penicillin/streptomycin (Gibco). The FaDu cell line was maintained in RPMI1640 (Gibco, Invitrogen) supplemented with 10% FBS. The Chk1/2 inhibitor prexasertib (Eli Lilly) was used at 1 nmol/L inUM-SCC1 and UM-SCC47 and at 10 nmol/LinUM-SCC2, UM-SCC6, UPCI:SCC090, and FaDu in vitro, and 4 mg/kg in vivo. Cetuximab (C225, Bristol Myers Squibb) was used at 0.25 μg/mL in vitro and 0.1 mg/injection in vivo.

### Cell proliferation

Cell proliferation assays were performed as described previously (15). Briefly, cells were seeded in 24-well plates and harvested at 48, 72, and 96 hours after treatments. Cells were washed with PBS, trypsinized, and diluted 1:20 in isotonic saline solution (RICCA Chemical, catalog #7210-5). Diluted cells were counted using a Beckman Z1 Coulter particle counter. Cell counts were represented as cells/mL.

### Colony formation assay

Clonogenic survival was assessed by colony formation assay as described previously (16). Cells were treated accordingly and remained undistributed for two weeks. Media were not replaced throughout the experiment. Cells were fixed and stained in 25% glutaraldehyde/12 mmol/L crystal violet solution and the numbers of colonies were counted. Survival fraction was calculated as follows: (number of colonies counted in experimental plate/number of cells seeded in experimental plate)/(number of colonies counted in control plate/number of cells seeded in control plate). Experiments were performed at least in triplicate.

### Apoptosis

Apoptosis was analyzed using the Annexin V-FITC Apoptosis Detection kit (BioVision Research Products, 3K101-400) according to the manufacturer's instructions and was previously described (16).

### Protein expression

Protein was analyzed by SDS-PAGE as previously described (16). The following primary antibodies from Cell Signaling Technology were used at manufacturer-recommended dilutions for immunoblotting: cleaved caspase-3 (#9661), total caspase 3 (#9668), cleaved caspase-9 (#9501), total caspase-9 (#9502), phospho-Chk1 (Ser296; #2349), total Chk1 (#2360), phos-pho-Chk2 (Thr68; #2661), total Chk2 (2662), and γH2AX (#9718). β-Actin (Santa Cruz Biotechnology, catalog #sc-47778) was included as a loading control. Species-specific horseradish peroxidase-conjugated secondary antibodies (Santa Cruz Biotechnology) were used at 1:20,000 dilution.

### Cell cycle

Cell-cycle distribution was measured as previously described (17). Cells were seeded in 100 mm^2^ dishes and treated accordingly. 48 and 72 hours after treatments, cells were collected, fixed, treated with RNAse (Sigma, catalog # R-4875), stained with propidium iodide (PI), and read on FACS Calibur using Cell Quest. Data were analyzed using ModFit LT (Verity Software Inc).

### Animal studies

All animal procedures were approved and in accordance with the UAB Institutional Animal Care and Use Committee guidelines. Four-week-old, 20 g, female athymic nude mice (Charles River Laboratories) were allowed to acclimatize for 1 week before experiments. For the orthotopic UM-SCC1-luc model, 100,000 cells were injected into the oral tongue, and tumors were imaged biweekly using a luciferase bioluminescence assay starting at day 4 after injection. Mice received intraperitoneal injections of D-luciferin substrate (150 mg/kg) 15 minutes before imaging, and luminescence was measured in photons per second. For the heterotopic UM-SCC47 model, 3 × 10^6^ cells were injected into the right flank, and tumors were measured by caliper biweekly starting at day 4 after injection. Mice bearing HNSCC cell line xenografts were subjected to 3 weekly cycles of prexasertib (Mondays and Thursdays), cetuximab (Mondays), and 2 Gy irradiation (Mondays and Thursdays). Prexasertib was injected subcutaneously at 4 mg/kg twice a day. Cetuximab was given at 0.1 mg/injection intraperitoneally. Twenty percent Captisol was used as a vehicle control.

### Statistical analysis

Data were analyzed by ANOVA followed by Bonferroni post-test using GraphPad Prism version 4.02 (GraphPad Software). Data are presented as average ± SE.

## Results

### Combined prexasertib with cetuximab and IR decreases cell proliferation

**Figure 1:**
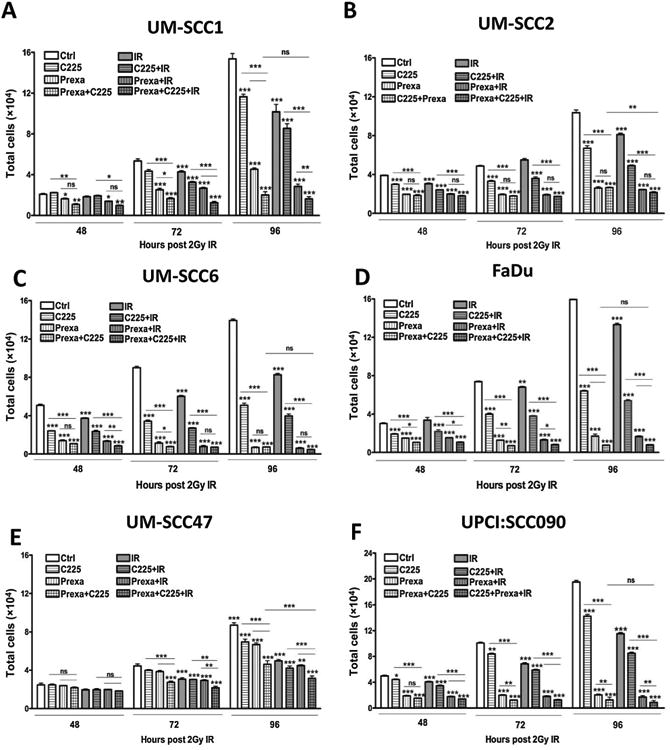
Combination treatment with prexasertib, cetuximab, and IR decreases cell proliferation. UM-SCC1 (**A**), UM-SCC2 (**B**), UM-SCC6 (**C**), FaDu (**D**), UM-SCC47 (**E**), and UPCI:SCC090 cells (**F**) were treated with either vehicle or 0.25 μg/mL C225 for 16 hours, then 1 or 10 nmol/L prexasertib (prexa) for 2 hours, followed by sham or 2 Gy IR. Cell numbers were counted at 48, 72, and 96 hours after IR using the Beckman Z1 Coulter particle counter. Shown is the mean ± SEM from one of two independent experiments performed in triplicate;*, *P* < 0.05; **, *P* < 0.01; ***, *P* < 0.001.

To evaluate the antitumor effects of prexasertib with cetuximab and IR, we first assessed cell proliferation following various combinations of prexasertib, cetuximab (C225), and IR in HNSCC cell lines. In both HPV-positive and -negative cell lines, treatment with C225 alone, prexasertib alone, or combination of C225 plus prexasertib with or without IR significantly decreased proliferation compared with control at all timepoints (Fig. 1A–F). Also, in most cell lines tested (UM-SCC1, UM-SCC6, FaDu, UM-SCC47 and UPCI:SCC090 cells), the addition of prexasertib to C225 further reduced cell proliferation compared with single-agent alone at the 72- and 96-hour time points. Similarly, in all cell lines tested, combining prexasertib with C225 and IR decreased cell proliferation more so compared with IR alone with or without C225 at both 72- and 96-hour timepoints. Interestingly, in UM-SCC1, UM-SCC6, FaDu, and UPCI:SCC090 cells, prexasertib with C225 reduced cell proliferation to a similar extent as the triple combination. These results suggest that prexasertib exerts anti-proliferative effects against head and neck cancer cells and that combining prexasertib with C225 and/or IR results in further suppression of cancer cell growth.

Next, we also assessed cell survival with different doses of IR using the colony formation assay in the UM-SCC1 and UM-SCC47 cells treated with prexasertib and C225. Similar to the cell proliferation data, combined prexasertib with C225 significantly reduced cell survival fraction compared with either agent alone in both cell lines (Supplementary Fig. S1A and S1B). In the HPV-negative UM-SCC1 cells but not the HPV-positive UM-SCC47 cells, the addition of prexasertib also improved the effectiveness of IR alone or IR with C225 (Supplementary Fig. S1C and S1D). These results suggest that prexasertib induces cytotoxicity in HNSCC cells and that combination treatment of prexasertib, C225, and IR may be effective in inhibiting HNSCC cell growth.

### Prexasertib with cetuximab and IR enhances apoptosis and generates persistent DNA damage

**Figure 2:**
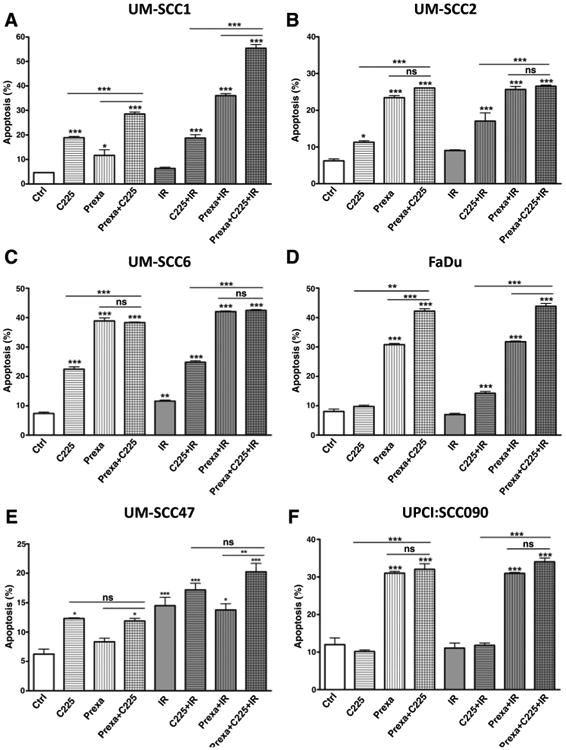
Prexasertib with cetuximab and IR induces early apoptosis. Annexin V apoptotic assay in UM-SCC1 (**A**), UM-SCC2 (**B**), UM-SCC6 (**C**), FaDu (**D**), UM-SCC47 (**E**), and UPCI:SCC090 cells (**F**). Cells were treated with either vehicle or 0.25 mg/mL C225 for 16 hours, then 1 or 10 nmol/L prexasertib (prexa) for 2 hours, followed by sham or 2 Gy IR. Cells were harvested at 48 hours after IR and evaluated for Annexin V positivity by flow cytometry. Shown is the mean ± SEM from one of two independent experiments performed in triplicate; *, *P* < 0.05; **, *P* < 0.01; ***, *P* < 0.001.

To investigate the mechanism of cytotoxicity of combining prexasertib, C225, and IR, we first examined cells for Annexin V, an early cell surface marker of apoptosis, 48 hours after treatment. In both HPV-negative and HPV-positive cell lines, treatment with C225 alone, prexasertib alone, or C225 and prexasertib all increased apoptosis compared with control (Fig. 2A–F). In both UM-SCC1 and UM-SCC47 cells, there was the greatest induction of apoptosis with the triple combination of prexasertib, C225, and IR. In the remaining cell lines, variable induction of apoptosis was observed, but in general, treatment groups containing prexasertib yielded greater apoptosis compared with those without prexasertib.

**Figure 3:**
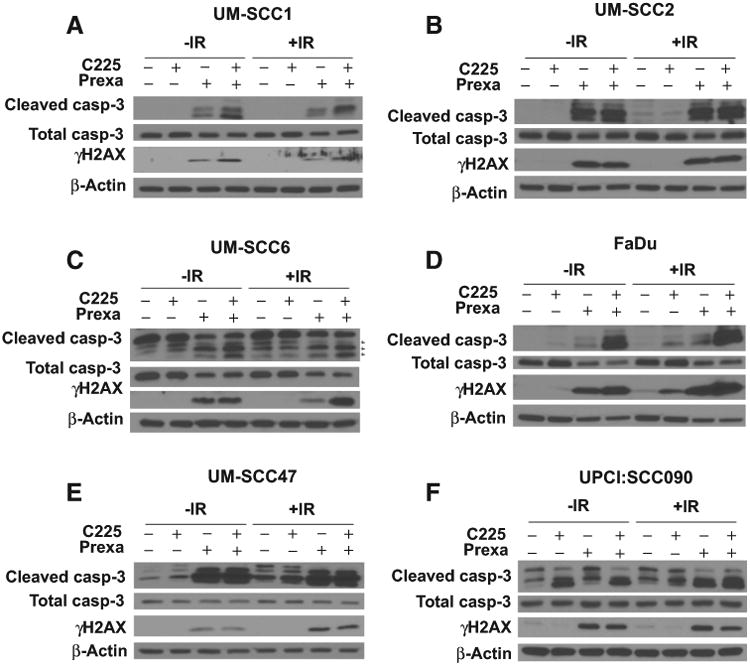
Prexasertib with cetuximab and IR increases caspase-3 cleavage and induces persistent DNA damage. UM-SCC1 (**A**), UM-SCC2 (**B**), UM-SCC6 (**C**), FaDu (**D**), UM-SCC47 (**E**), and UPCI:SCC090 cells (**F**) were treated with either vehicle or 0.25 μg/mL C225 for 16 hours, then 1 or 10 nmol/L prexasertib (prexa) for 2 hours, followed by sham or 2 Gy IR. Cell lysates were harvested at 48 hours after IR and analyzed by Western blot analysis for total and cleaved caspase-3 and γ-H2AX protein induction. Shown are representative blots from at least two independent experiments, using β-actin as a loading control.

To confirm the increased apoptotic signaling, we also investigated caspase-3 cleavage in treated cells. In the HPV-negative cells, there is minimal caspase-3 cleavage observed at 48 hours following low dose (2 Gy) of IR. In contrast, a robust increase in cleaved caspase-3 was observed following treatment with prexasertib alone or in combination with C225 or IR or both C225 and IR (Fig. 3A–D). A similar trend was observed in the HPV-positive cell lines (Fig. 3E and F).

As apoptosis can be activated by the presence of persistent DNA damage, a known effect of CHKi treatment, we assessed phosphorylation of H2AX (γH2AX), a well-accepted marker of DNA double strand breaks. In all cell lines, γH2AX remained detectable up to 48 hours after treatment with prexasertib or combination C225 and prexasertib with and without IR (Fig. 3A–F). In UM-SCC1, UM-SCC6, and FaDu cells, γH2AX protein induction was slightly higher in cells treated with C225 plus prexasertib compared with prexasertib alone, although this effect was not seen in UM-SCC47, UPCI:SCC090, or UM-SCC2 cells. In contrast, persistent γH2AX was not observed in the cells treated with low-dose of C225 alone. Taken together, these findings suggest that combination of C225 and prexasertib with or without IR activates caspase cleavage and increases cell apoptosis to a greater extent than either agent alone, possibly as a result of persistent DNA damage.

### Prexasertib induced S-phase accumulation in cells

**Figure 4:**
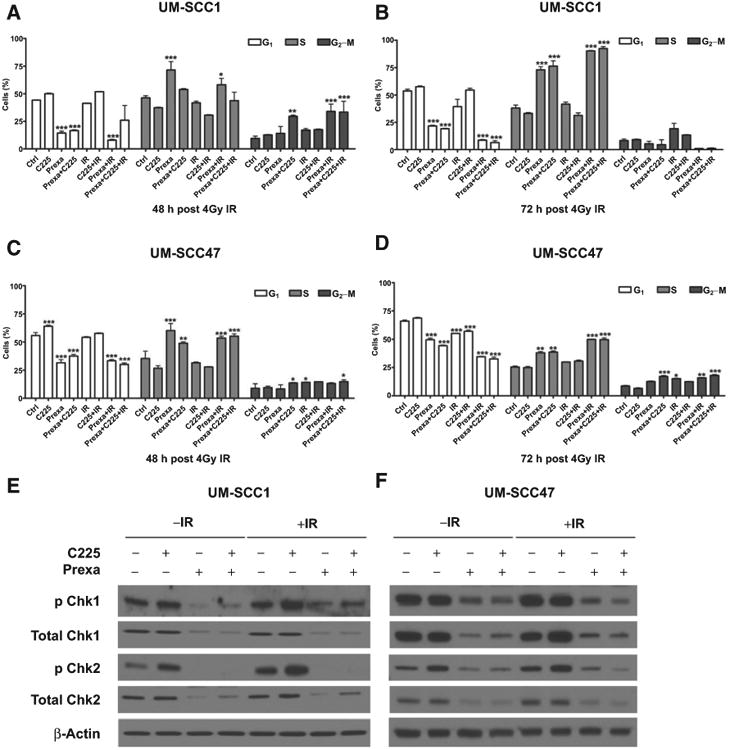
Prexasertib induces S-phase accumulation in cells and abrogates cetuximab and IR induced checkpoint activation. **A-D**, UM-SCC1 and UM-SCC47 cells were treated with either vehicle or 0.25 μg/mL C225 for 16 hours, then 1 nmol/L prexasertib (prexa) for 2 hours, followed by sham or 4 Gy IR. Cells were stained with propidium iodide (Pi) at (**A** and **C**) 48 or (**B** and **D**) 72 hours after IR and analyzed for cell-cycle distribution by flow cytometry. Shown is the mean ± SEM from one of three independent experiments performed in triplicate; *, *P* < 0.05; **, *P* < 0.01; ***, *P* < 0.001. UM-SCC1 (**E**) and UM-SCC47 (**F**) cells were treated, and cell lysates were harvested at 48 hours after IR and assessed for expression of Chk1 and Chk2, including both total and phosphorylated protein levels, by Western blot analysis. β-Actin was used as a loading control. Shown are representative blots from at least two independent experiments.

Martinelli and colleagues (18) and King and colleagues (14) recently reported that prexasertib as a CHKi alters cell-cycle distribution in treated cells. To further evaluate the effect of prexasertib on cell-cycle distribution of treated cells, we analyzed cell cycle by flow cytometry. First, as a control, we assessed the ability of prexasertib to circumvent cell-cycle alteration from serum starvation. We found that prexasertib reduced G_1_ phase accumulation due to serum starvation (Supplementary Fig. S2). Next, we analyzed cell-cycle distribution in UM-SCC1 and UM-SCC47 cells following various treatments. Consistent with previous data (14, 18), prexasertib-treated groups had reduced the percentage of cells in G_1_ phase. Accordingly, an increased percentage of cells in the S-phase was observed as compared with groups not treated with prexasertib (Fig. 4A–D). These results indicate that prexasertib increases S-phase accumulation of treated cells, which may be due to the accumulation of DNA damage that cannot be resolved during DNA replication leading to cell death.

### Prexasertib abrogates cetuximab- and IR-induced checkpoint activation

Our rationale for combining prexasertib with C225 and IR was that prexasertib would inhibit the cell-cycle checkpoint response to genomic stress mediated by cetuximab and/or IR and therefore block a potential mechanism for therapeutic resistance. Thus, we next evaluated the effects of prexasertib, C225, and IR on checkpoint signaling. We used Western blot analysis to compare total and phosphorylated protein levels of Chk1 and Chk2 in HNSCC cells following treatment. In UM-SCC1 cells, C225 induced an increase inChk1 and Chk2 phosphorylation, whereas IR increased Chk2 phosphorylation (Fig. 4E). Baseline, C225-induced, and IR-induced phosphorylation ofChk1 and Chk2 was blocked by prexasertib, although we did not observe apparent differences in phosphorylation levels between prexasertib alone, C225 and prexasertib, and C225 with prexasertib and IR(Fig. 4E). Interestingly, total Chk1 and Chk2 expression was decreased to the same extent as phosphorylated Chk1 and Chk2 in prexasertib-treated UM-SCC1 cells (Fig. 4E).

In contrast, UM-SCC47 cells had high expression of total and phosphorylated Chk1 at baseline, which was not further increased by C225 or IR. However, elevated phospho-Chk2 was observed following C225 treatment (Fig. 4F). Baseline and C225-induced Chk1/2 phosphorylation was decreased by prexasertib, although the triple combination of C225, prexasertib and IR was superior to prexasertib alone and C225 with prexasertib in these cells (Fig. 4F). Again, total Chk1 and Chk2 expression was reduced by prexasertib to the same degree as phospho-Chk1 and phospho-Chk2 (Fig. 4F). These results show that C225-induced checkpoint activation in both UM-SCC47 and UM-SCC1 HNSCC cells, as well as IR-induced checkpoint activation in UM-SCC1 cells, were effectively inhibited by prexasertib. Our data also indicate that prexasertib decreases total Chk1/2 protein expression.

### Prexasertib plus cetuximab-IR increases tumor growth delay in HNSCC xenografts in vivo

**Figure 5:**
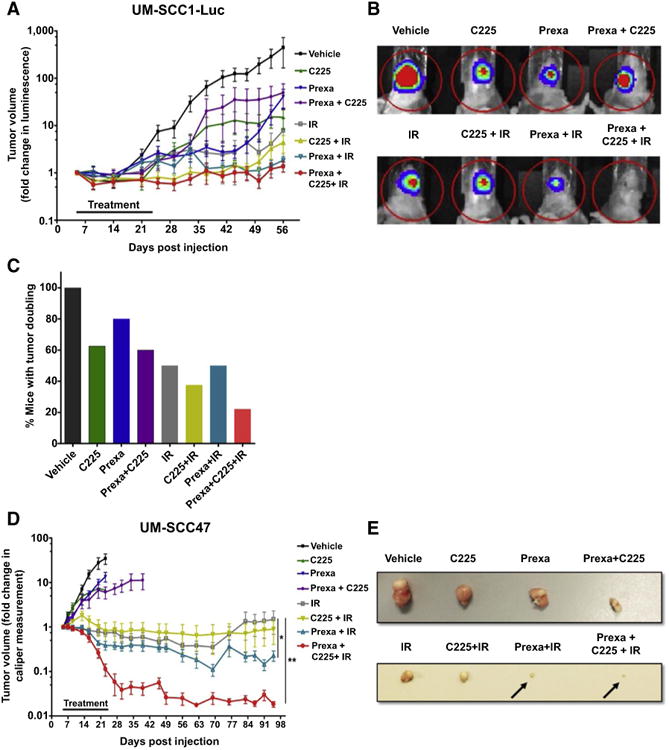
Prexasertib plus cetuximab-IR delays tumor growth of HPV-negative UM-SCC1-Luc orthotopic xenografts and HPV-positive UM-SCC47 heterotopic xenografts. **A-D**, The tongues of athymic nude mice were injected with UM-SCC1 luciferase-expressing cells (UM-SCC1-Luc), and tumor volume was measured by bioluminescence imaging twice weekly. **A**, UM-SCC1 tumor growth over time, normalized to luminescence measurement at the start of treatment on day 5. Shown is the mean fold change in tumor volume ± SEM. *N* = 8-10 mice for all treatment groups. **B**, Representative optical images of UM-SCC1 tumor luminescence at day 56. **C**, The percentage of mice with a two fold increase or greater in UM-SCC1 tumor volume in each treatment group at day 56. **D-E**, The flanks of athymic nude mice were injected subcutaneously with UM-SCC47 cells, and tumor volumes were measured by digital caliper twice a week and calculated using the equation: (width×length×height)/2. **D**, UM-SCC47 tumor growth over time, normalized to caliper measurement at the start of treatment on day 5. Shown is the mean fold change in tumor volume ± SEM. *N* = 8-10 mice for all treatment groups; *, *P* < 0.05, IR versus prexa + IR; **, *P* <0.01, IR versus prexa + C225 + IR. **E**, Representative images of harvested UM-SCC47 tumors for each treatment group. Vehicle, C225, prexasertib, and C225 + prexasertib were harvested at day 20. IR, IR + C225, IR + prexasertib, IR + C225 + prexasertib were harvested at day 49.

To validate our in vitro data demonstrating the potential activity of prexasertib in combination with C225 and IR in HNSCC cells, we measured in vivo tumor growth delay in mice bearing orthotopic UM-SCC1-luciferase or heterotopic UM-SCC47 xenografts. First, a pilot study was performed to assess the tolerability and toxicity of combining prexasertib with C225 and IR in UM-SCC1-luciferase cells (UM-SCC1-Luc). As the order and timing of dosing are thought to influence the efficacy of combination treatment regimens, especially those including EGFR inhibition, we also used the pilot study to explore four different dosing schedules based on possible mechanisms of synergy between prexasertib, C225, and IR (Table 1; ref. 19). No significant weight losses were observed in any of the treatment groups (Supplementary Fig. S3A). Although we observed similar tumor growth suppression at 75 days in mice receiving triple combination therapy using treatment schedules 2 and 4 (Table 1; Supplementary Fig. S3B), schedule 2 (prexasertib concurrent with C225 and IR) had a slightly better response rate at day 100 and, accordingly, we continued with this strategy in all subsequent experiments. In a repeat experiment using 10 mice per group, we saw significant tumor growth delay in all treatment groups as compared with vehicle (Fig. 4A and B). Although the differences between treatment groups were not statistically significant, mean fold change in tumor volume was smallest in mice treated with combination of prexasertib, C225, and IR (Fig. 5A). This was also apparent in the representative tumor volumes as depicted by luciferase activity (Fig. 5B). Furthermore, we observed a significantly higher percentage of “responders” in the triple combination group as compared with the other treatment groups based on the percentage of mice with a 2-fold increase in tumor volume (Fig. 5C), with only 22.2% of mice in the triple combination group experiencing tumor doubling compared with other treatment groups, such as prexasertib with C225 (62.5%) or prexasertib with IR (50%). Similar results were also observed when we analyzed the percentage of mice with tumor quadrupling (Supplementary Fig. S3C).

A similar experiment was also performed using the HPV-positive UM-SCC47 heterotopic flank model. In these cell line xenografts, we saw a substantial tumor growth delay in all treatment groups with IR as expected. Interestingly, the combination of prexasertib, C225, and IR significantly inhibited tumor growth as compared with other treatment groups, as shown in the tumor growth delay graph (Fig. 5D) and representative tumor images (Fig. 5E). Importantly, combination therapy did not cause excess toxicity as assessed by body weight (Supplementary Fig. S3D– S3E). These results support the enhanced in vivo growth suppression in response to combination treatment of prexasertib, C225 and IR without apparent significant toxicities in HNSCC.

## Discussion

Although targeted therapies against EGFR, such as C225, have been developed for use in HNSCC, resistance is a common occurrence and survival rates remain poor. Therefore, effective alternative treatments are greatly needed to improve clinical outcomes in this disease. In this study, we demonstrate that prexasertib, an inhibitor of Chk1/2, attenuates checkpoint activation induced by C225 and IR, leading to persistent DNA-damage and increased apoptotic cell death in both HPV-positive and HPV-negative HNSCC cell lines. Moreover, combining prexasertib with C225 and IR led to a significant tumor growth delay in mice bearing orthotopic or heterotopic HNSCC xenografts. Thus, combining prexasertib with C225 and IR may be an innovative treatment strategy for both HPV-positive and HPV-negative HNSCC patients.

We found that prexasertib treatment in HNSCC cells resulted in S-phase accumulation and induction of persistent γ-H2AX, suggesting that the induction of replication stress may lead to the cell death observed in treated cells. These results are similar to those reported in recent studies from Martinelli and colleagues (18) and King and colleagues (14), which showed induced replication catastrophe by checkpoint inhibition monotherapy. However, in our study, especially in the context of combination therapy, other mechanisms of cell death such as mitotic catastrophe cannot be ruled out.

Combination treatment with prexasertib, C225, and IR was also sufficient to overcome the underlying variability in cell-cycle checkpoint pathways (20, 21), leading to a significant decrease in survival in vitro and sustained tumor growth delay in vivo in both HPV-positive and -negative HNSCC cells. These results suggest that combined treatment with EGFRi and CHKi and IR may be a broadly applicable therapeutic strategy for HNSCCs.

Decreased phosphorylation of checkpoint proteins in response to CHKi was somewhat expected. P-Chk1(Ser296) detects autophosphorylation, which should be directly inhibited by prexasertib, and P-Chk2(Thr68) detects phosphorylation by ATM/ATR, which may be decreased because altered checkpoints affect the ability of cells to activate the DNA damage response. Consistent with our findings, it has been shown that radiotherapy combined with CHKi reduces homologous DNA repair in pancreatic and breast cancer models (10, 11). However, we were surprised to observe reduced total protein expression of Chk1 and Chk2 in HNSCC cells treated with prexasertib. This phenomenon was observed in both UM-SCC1 and UM-SCC47 cells. Upon further investigation, our results are also consistent with Supplementary Data from King and colleagues(14), where prexasertib produced a dose-dependent decrease in total protein expression of Chk1 and Chk2.

Previous studies have demonstrated that phosphorylation of Chk1/2 causes a conformational change, which activates kinase function while simultaneously exposing a ubiquitination site which, allows for protein degradation (22). This negative regulatory mechanism provides a means of terminating the checkpoint once the activation stress has been removed, and, accordingly, the active conformation of Chk1/2 is much more unstable than the closed/inactive state. As prexasertib is a competitive inhibitor that occupies the ATP-binding domain of Chk1 and 2, the drug may induce a similar conformation change to the active state, resulting in protein destabilization and eventual degradation. Alternatively, the effects of prexasertib on Chk1/2 total protein expression may be related to inhibition of downstream checkpoint signaling, including the recruitment and activation of proteins that repair DNA damage. Some DNA repair proteins, including DNA-Pk_CS_ and Metnase, have a secondary role in checkpoint stabilization, and decreased recruitment may repress this positive feedback loop (23, 24). It is unclear how the effects of prexasertib on total protein expression compared with blockade of autophosphorylation with respect to anticancer activity or potential adverse side effects.

One of the factors limiting the use of combination therapies, and specifically combinations of targeted therapies, are contraindications, including comorbidities and adverse or allergic responses. For example, some patients have IgE-mediated hyper-sensitivity to EGFR inhibitors, including C225 and panitumumab, leading to severe infusion reactions that can ultimately be fatal (25, 26). The prevalence of these reactions is highly variable, ranging from 3% to 20%, and is related to previous allergy history which, in turn, differs by geographic region (27). In this study, dual therapy with prexasertib plus IR consistently matched the cytotoxicity of C225 plus IR in both HPV-negative and HPV-positive HNSCC cells in in vitro and in vivo assays. Furthermore, in some of the cell lines tested, prexasertib plus IR treatment had similar antitumor effects as triple combination treatment. These data suggest that prexasertib, when given with IR, may be an appropriate alternative treatment for HNSCC patients not eligible for C225 or cisplatin. Additional in vivo and clinical studies are needed to rigorously test this hypothesis.

An interesting observation from our in vitro study was that the cytotoxicity observed with prexasertib and C225 was comparable with the triple combination (prexasertib, C225, and IR) in some of the tested cell lines. However, in the in vivo studies, the triple combination exhibited greater antitumor effects compared with the double combination of prexasertib and C225. This may be related to the inherent shortcomings of the in vitro model that demonstrates the short-term effects of the tested therapies, because the in vitro models does not account for the accumulated long-term effects of the combination therapy observed in the in vivo models. Even modest changes in the rate of cytotoxicity may over time contribute to significant reductions in tumor volumes in vivo. Nevertheless, the combination of prexasertib and C225 may be an interesting therapeutic strategy, which is currently being tested in a clinical trial (NCT02124148) for patients with recurrent head and neck cancer.

The current non-surgical standard therapies for locally advanced HNSCC are concurrent C225 with IR and cisplatin with IR. Cisplatin induces DNA damage by forming DNA adducts, which therefore activate the cell cycle checkpoint response. It is interesting to test whether combining prexasertib with cisplatin-IR will also enhance cytotoxicity in HNSCC. Overall, our findings from this study support further clinical investigation of prexasertib in locally advanced HNSCC to improve response and reduce acquired resistance in patients treated with C225 and IR. A phase Ib clinical trial to test prexasertib in combination with C225 and irradiation in patients with locally advanced HNSCC is currently ongoing (NCT02555644).

## Supplementary Material

Supplemental Fig 1

Supplemental Fig 2

Supplemental Fig 3

Supplemental Figure Legend

## Figures and Tables

**Table 1:** In vivo dosing schedules for prexasertib (P) + C225 + IR

	Monday		Tuesday		Wednesday		Thursday		Friday	
					
Schedule	AM	PM	AM	PM	AM	PM	AM	PM	AM	PM
1	C225–IR		P	P	P	P	P–IR	P		
2	P+C225–IR	P					P–IR	P		
3	C225–IR		P	P			IR		P	P
4	P+C225–IR	P	P	P			P–IR	P	P	P

## References

[1] Chaturvedi AK, Engels EA, Pfeiffer RM, Hernandez BY, Xiao W, Kim E (2011). Human papillomavirus and rising oropharyngeal cancer incidence in the United States. J Clin Oncol.

[2] Ang KK, Harris J, Wheeler R, Weber R, Rosenthal DI, Nguyen-Tan PF (2010). Human papillomavirus and survival of patients with oropharyngeal cancer. N Engl J Med.

[3] Fakhry C, Westra WH, Li S, Cmelak A, Ridge JA, Pinto H (2008). Improved survival of patients with human papillomavirus-positive head and neck squamous cell carcinoma in a prospective clinical trial. J Natl Cancer Inst.

[4] Bonner JA, Harari PM, Giralt J, Azarnia N, Shin DM, Cohen RB (2006). Radiotherapy plus cetuximab for squamous-cell carcinoma of the head and neck. N Engl J Med.

[5] Chung CH, Ely K, McGavran L, Varella-Garcia M, Parker J, Parker N (2006). Increased epidermal growth factor receptor gene copy numberis associated with poor prognosis in head and neck squamous cell carcinomas. J Clin Oncol.

[6] Huang SM, Harari PM (2000). Modulation of radiation response after epidermal growth factor receptor blockade in squamous cell carcinomas: inhibition of damage repair, cell cycle kinetics, and tumor angiogenesis. Clin Cancer Res.

[7] Nowsheen S, Bonner JA, Lobuglio AF, Trummell H, Whitley AC, Dobelbower MC (2011). Cetuximab augments cytotoxicity with poly (adp-ribose) polymeraseinhibition in head and neck cancer. PLoS ONE.

[8] Benavente S, Huang S, Armstrong EA, Chi A, Hsu KT, Wheeler DL (2009). Establishment and characterization of a model of acquired resistance to epidermal growth factor receptor targeting agents in human cancer cells. Clin Cancer Res.

[9] Frederick MJ, VanMeter AJ, Gadhikar MA, Henderson YC, Yao H, Pickering CC (2011). Phosphoproteomic analysis of signaling pathways in head and neck squamous cell carcinoma patient samples. Am J Pathol.

[10] Al-Ejeh F, Pajic M, Shi W, Kalimutho M, Miranda M, Nagrial AM (2014). Gemcitabine and CHK1 inhibition potentiate EGFR-directed radioimmunotherapy against pancreatic ductal adenocarcinoma. Clin Cancer Res.

[11] Morgan MA, Parsels LA, Zhao L, Parsels JD, Davis MA, Hassan MC (2010). Mechanism of radiosensitization by the Chk1/2 inhibitor AZD7762 involves abrogation of the G2 checkpoint and inhibition of homologous recombinational DNA repair. Cancer Res.

[12] Al-Ejeh F, Shi W, Miranda M, Simpson PT, Vargas AC, Song S (2013). Treatment of triple-negative breast cancer using anti-EGFR-directed radioimmunotherapy combined with radiosensitizing chemotherapy and PARP inhibitor. J Nucl Med.

[13] McNeely S, Beckmann R, Bence Lin AK (2014). CHEK again: revisiting the development of CHK1 inhibitors for cancer therapy. Pharmacol Ther.

[14] King C, Diaz HB, McNeely S, Barnard D, Dempsey J, Blosser W (2015). LY2606368 causes replication catastrophe and antitumor effects through CHK1-dependent mechanisms. Mol Cancer Ther.

[15] Weaver AN, Burch MB, Cooper TS, Della Manna DL, WeiS, Ojesina AI (2016). Notch signaling activation is associated with patient mortality and increased FGF1-mediated invasion in squamous cell carcinoma of the oral cavity. Mol Cancer Res.

[16] Nowsheen S, Cooper T, Stanley JA, Yang ES (2012). Synthetic lethal interactions between EGFR and PARP inhibition in human triple negative breast cancer cells. PLoS ONE.

[17] Feng Z, Kachnic L, Zhang J, Powell SN, Xia F (2004). DNA damage induces p53-dependent BRCA1 nuclear export. J Biol Chem.

[18] Di Rorá AGL, Iacobucci I, ImbrognoE, Papayannidis C, Derenzini E, Ferrari A (2016). Prexasertib, a Chk1/Chk2 inhibitor, increases the effectiveness of conventional therapy in B-/T- cell progenitor acute lymphoblastic leukemia. Oncotarget.

[19] Nyati MK, Morgan MA, Feng FY, Lawrence TS (2006). Integration of EGFR inhibitors with radiochemotherapy. Nat Rev Cancer.

[20] Chaurushiya MS, Weitzman MD (2009). Viral manipulation of DNA repair and cell cycle checkpoints. DNA Repair.

[21] Galloway DA, McDougall JK (1996). The disruption of cell-cycle checkpoints by papillomavirus oncoproteins contributes to anogenital neoplasia. Semin Cancer Biol.

[22] Zhang YW, Brognard J, Coughlin C, You Z, Dolled-Filhart M, Aslanian A (2009). The F box protein Fbx6 regulates Chk1 stability and cellular sensitivity to replication stress. Mol Cell.

[23] Williamson EA, Wu Y, Singh S, Byrne M, Wray J, Lee SH (2014). The DNA repair component Metnase regulates Chk1 stability. Cell Div.

[24] Lin YF, Shih HY, Shang Z, Matsunaga S, Chen BP (2014). DNA-PKcs is required to maintain stability of Chk1 and Claspin for optimal replication stress response. Nucleic Acids Res.

[25] George TJ, Laplant KD, Walden EO, Davis AB, Riggs CE, Close JL (2010). Managing cetuximab hypersensitivity-infusion reactions: incidence, risk factors, prevention, and retreatment. J Support Oncol.

[26] Chung CH, Mirakhur B, Chan E, Le QT, Berlin J, Morse M (2008). Cetuximab-induced anaphylaxis and IgE specific for galactose-alpha-1,3-galactose. N Engl J Med.

[27] O'Neil BH, Allen R, Spigel DR, Stinchcombe TE, Moore DT, Berlin JD (2007). High incidence of cetuximab-related infusion reactions in Tennessee and North Carolina and the association with atopic history. J Clin Oncol.

