# Supplementary figures and images for: Combining Chk1/2 Inhibition with Cetuximab and Radiation Enhances In Vitro and In Vivo Cytotoxicity in Head and Neck Squamous Cell Carcinoma
Source: Mol Cancer Ther. Author manuscript; Available in PMC 2018 Apr 1. (PMC5560482; doi:10.1158/1535-7163.MCT-16-0352)

## Slide 1
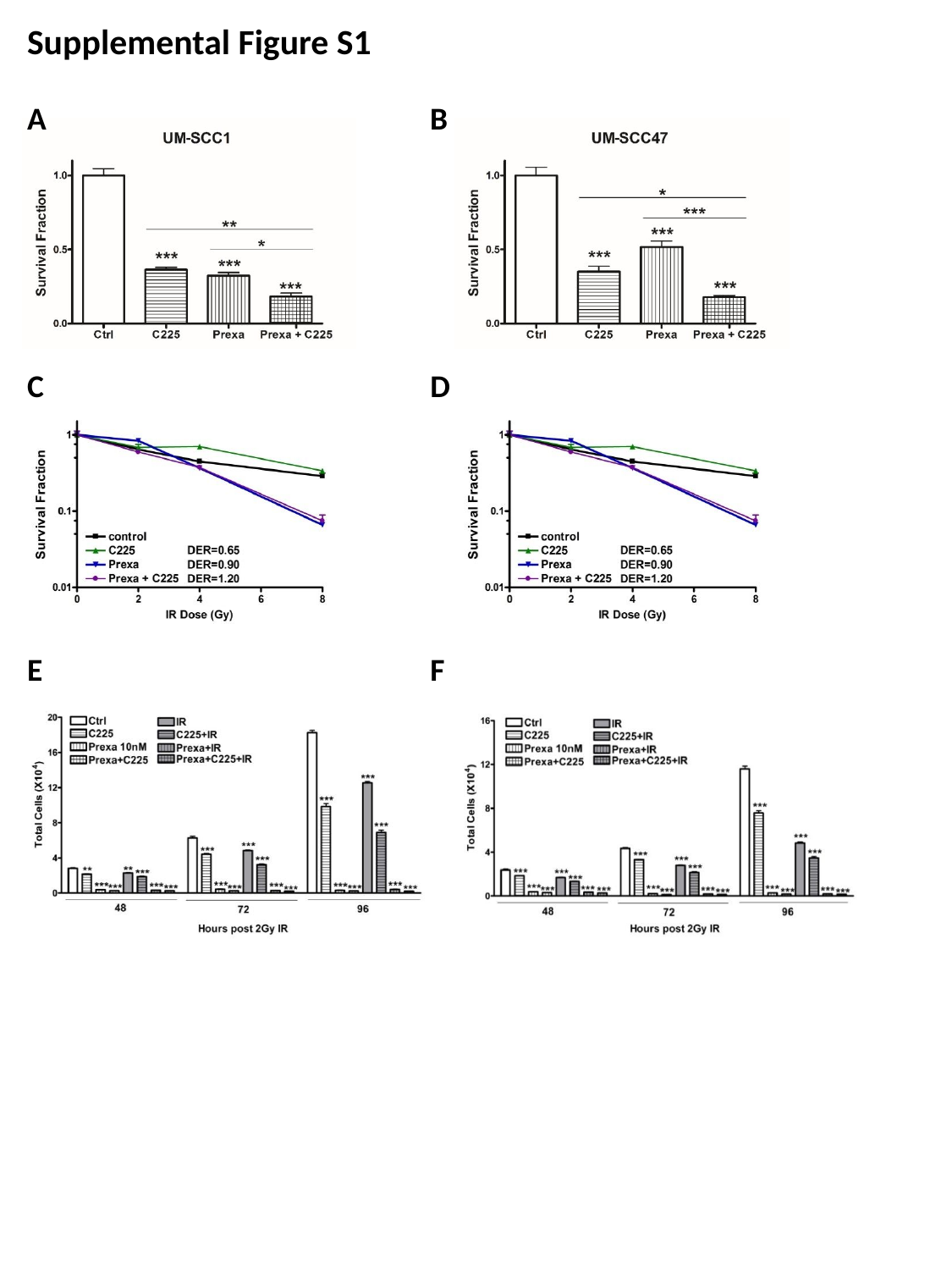

Supplemental Figure S1
A
B
C
D
E
F

Supplement: Supplemental Fig 1 [file NIHMS888327-supplement-Supplemental_Fig_1.pptx]

## Slide 1
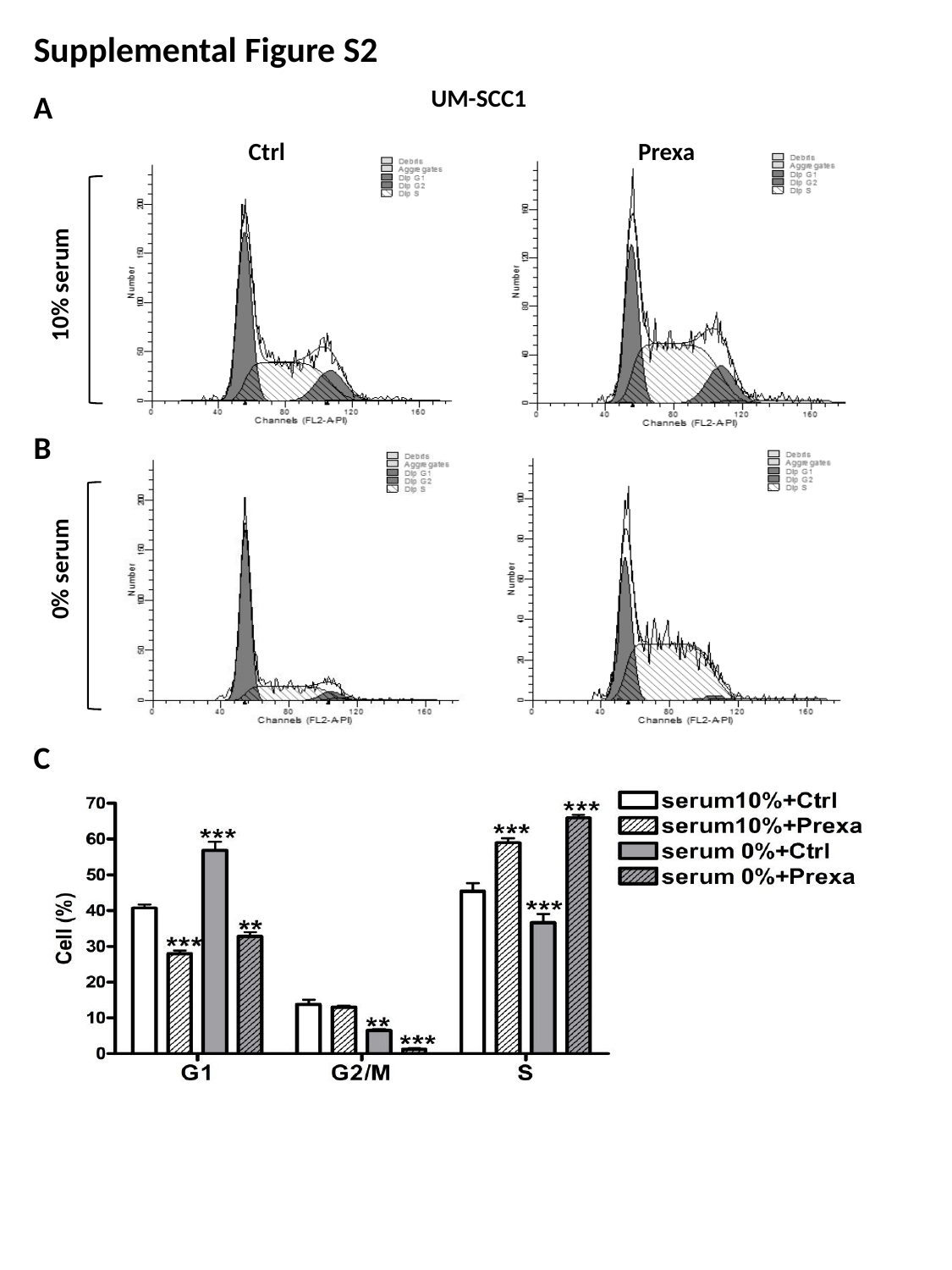

Supplemental Figure S2
UM-SCC1
A
Ctrl
Prexa
10% serum
B
0% serum
C

Supplement: Supplemental Fig 2 [file NIHMS888327-supplement-Supplemental_Fig_2.pptx]

## Slide 1
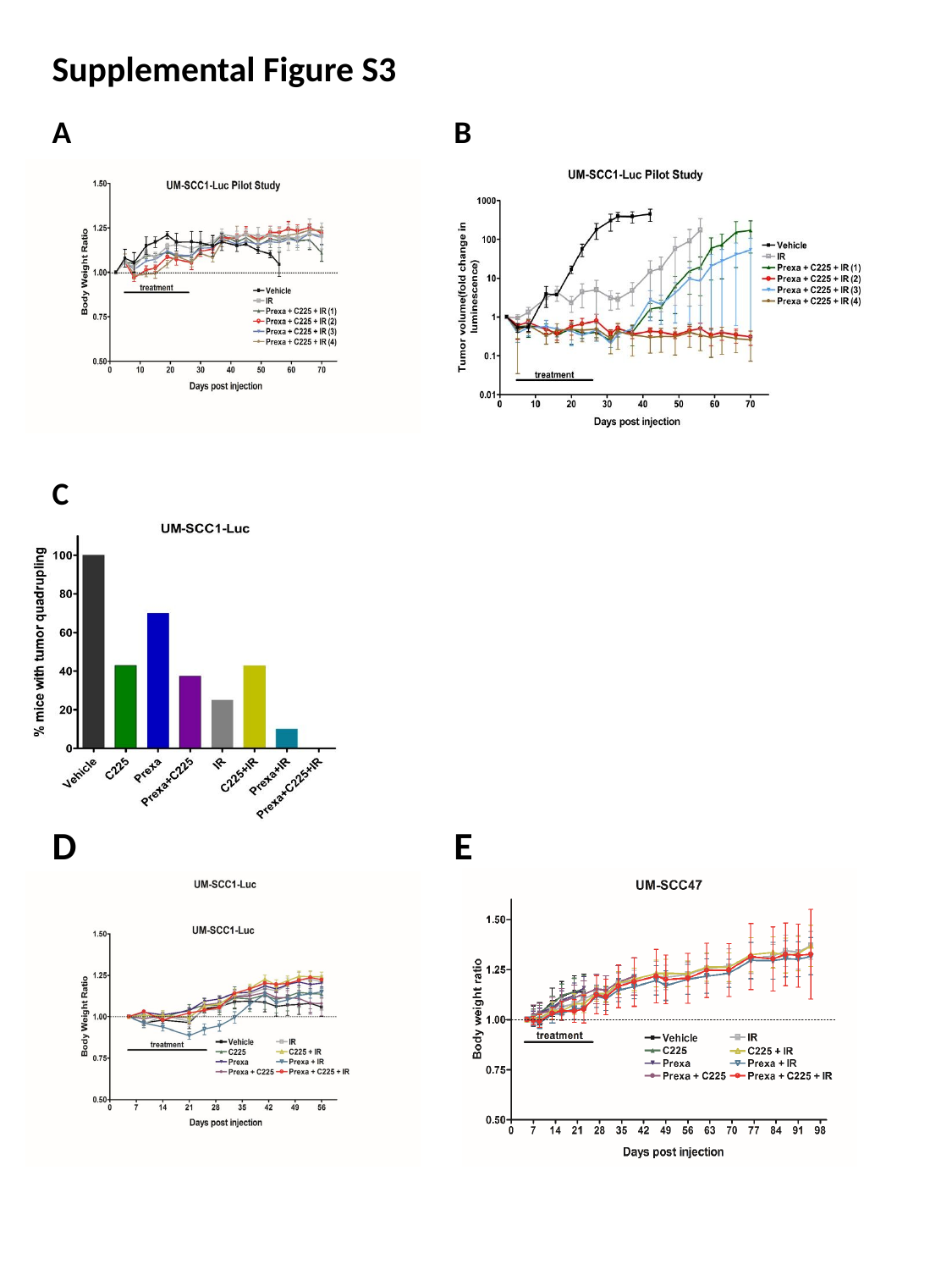

Supplemental Figure S3
A
B
C
D
E

Supplement: Supplemental Fig 3 [file NIHMS888327-supplement-Supplemental_Fig_3.pptx]
