# Supplementary material for: Combining Chk1/2 Inhibition with Cetuximab and Radiation Enhances In Vitro and In Vivo Cytotoxicity in Head and Neck Squamous Cell Carcinoma
Source: Mol Cancer Ther. Author manuscript; Available in PMC 2018 Apr 1. (PMC5560482; doi:10.1158/1535-7163.MCT-16-0352)
Supplement: Supplemental Figure Legend [file NIHMS888327-supplement-Supplemental_Figure_Legend.pdf]

Supplemental Figure S1. Colony formation and cell proliferation assays in UM-SCC1 and UM-SCC47. Colony formation assay of UM-SCC1 and UM-SCC47 cells. (A-B) Cells were treated with vehicle or 0.25 µg/mL cetuximab (C225) for 16 hours and subsequently exposed to vehicle or 1nM prexasertib (prexa). (C-D) Cells were treated as before, 2 hours after vehicle or prexasertib, cells were subjected to various doses of IR. (E-F) Cells were treated with vehicle or 0.25 µg/mL cetuximab (C225) for 16 hours and subsequently exposed to vehicle or 10nM prexasertib (prexa), 2 hours after vehicle or prexasertib cells were counted by IR Beckman Z1 Coulter particle counter. Shown is the mean survival fraction (+/− SEM) from one of two independent experiments performed in triplicate. *p<0.05, **p<0.01, ***p<0.001.

Supplemental Figure S2. Cell cycle analysis in prexasertib treated UM-SCC1 cells in the presence or absence of serum. Cells were plated with 10% serum and then changed to 10% serum or serum free medium for 24 hours, followed by 1nM prexasertib (prexa) treatment. Cells were stained with Propidium Iodide after 24 hours prexasertib treatment and the cell cycle progression was evaluated by flow cytometry. (A) Representative cell cycle profile of 10% serum group with or without prexasertib. (B) Representative cell cycle profile of 0% serum group with or without perxasertib. (C) The cell cycle distribution analysis (G1, S and G2/M) is shown *p<0.05, **p<0.01, ***p<0.001. Shown is the mean +/- SEM from one of two independent experiments performed in triplicate.

Supplemental Figure S3. UM-SCC1-Luc *in vivo* pilot study and treatment toxicity as assessed by body weight in mice bearing HNSCC xenografts. (A) Body weight was recorded twice per week in mice bearing UM-SCC1-Luc orthotopic xenografts. Body weight ratio is

normalized to the original weight at the start of treatment on day 5. Shown is the mean body weight ratio +/- SEM, with n=3 mice for all treatment groups. The number in parentheses in the figure legends refers to dosing schedule as described in Table 1. (B) The effects of dosing schedule on tumor growth over time for UM-SCC1-luc orthotopic xenografts, normalized to luminescence measurement at the start of treatment on day 5. Shown is the mean fold change in tumor volume +/- SEM, with n=3 mice for each treatment group. The number in parentheses in the figure legends refers to dosing schedule as described in Table 1. Body weight was recorded twice per week in mice bearing. (C) The percentage of mice with a 4-fold increase or greater in UM-SCC1 tumor volume in each treatment group at day 56. (D) UM-SCC1-Luc orthotopic xenografts or (E) UM-SCC47 heterotopic xenografts and normalized to original weight at the start of treatment on day 5. Shown is the mean body weight ratio +/- SEM, with n=8-10 mice for all treatment groups.
